# Gene therapy for ocular hypertension using hfCas13d-mediated mRNA targeting

**DOI:** 10.1093/pnasnexus/pgaf168

**Published:** 2025-06-17

**Authors:** Siyu Chen, Zhiquan Liu, Chien-Hui Lo, Qing Wang, Ke Ning, Qi Zhang, Jingyu Zhao, Yingchun Shen, Yang Sun

**Affiliations:** Department of Ophthalmology, Stanford University School of Medicine, 1651 Page Mill Road, Rm 2220, Palo Alto, CA 94304, USA; Department of Ophthalmology, Stanford University School of Medicine, 1651 Page Mill Road, Rm 2220, Palo Alto, CA 94304, USA; Department of Ophthalmology, Stanford University School of Medicine, 1651 Page Mill Road, Rm 2220, Palo Alto, CA 94304, USA; Department of Ophthalmology, Stanford University School of Medicine, 1651 Page Mill Road, Rm 2220, Palo Alto, CA 94304, USA; Department of Ophthalmology, Stanford University School of Medicine, 1651 Page Mill Road, Rm 2220, Palo Alto, CA 94304, USA; Department of Ophthalmology, Stanford University School of Medicine, 1651 Page Mill Road, Rm 2220, Palo Alto, CA 94304, USA; Department of Ophthalmology, Stanford University School of Medicine, 1651 Page Mill Road, Rm 2220, Palo Alto, CA 94304, USA; Department of Medicine, John A. Burns School of Medicine, 651 Ilalo St, Honolulu, HI 96813, USA; Department of Ophthalmology, Stanford University School of Medicine, 1651 Page Mill Road, Rm 2220, Palo Alto, CA 94304, USA; Department of Ophthalmology, Palo Alto Veterans Administration, 3801 Miranda Ave, Palo Alto, CA 94305, USA

## Abstract

Glaucoma is a major global cause of irreversible vision loss. It is marked by elevated intraocular pressure (IOP) and the loss of retinal ganglion cells (RGC). While there are medical and surgical therapies for glaucoma aiming to reduce aqueous humor production or enhance its drainage, these treatments are often inadequate for effectively managing the disease. In this study, we developed a targeted therapy for glaucoma by knocking down two genes associated with aqueous humor production (aquaporin 1 [*AQP1*] and carbonic anhydrase type 2 [*CA2*]) using Cas13 RNA editing systems. We demonstrate that hfCas13d-mediated knockdown of *AQP1* and *CA2* significantly lowers IOP in wild-type mice and in a corticosteroid-induced glaucoma mouse model. We show that the lowered IOP results from decreasing aqueous production without affecting the outflow facility; this treatment also significantly promotes RGC survival as compared with untreated control groups. Therefore, CRISPR–Cas-based gene editing may be an effective treatment to lower IOP for glaucomatous optic neuropathy.

**Significance Statement**

In this study, we demonstrated that RNA-based gene editing can effectively knock down aquaporin 1 and carbonic anhydrase type 2 in mouse eyes, leading to a significant reduction in intraocular pressure in both normal mice and those with steroid-induced ocular hypertension. Our findings suggest that gene editing is a feasible therapeutic approach for glaucoma, a leading cause of blindness worldwide, and lay the groundwork for future clinical translation. This strategy offers several advantages, including eliminating the need for daily eye drop administration and allowing for flexible adjustment to accommodate patients with varying disease severity.

## Introduction

Glaucoma is a leading cause of progressive and irreversible blindness in the world. It is a group of optic neuropathies characterized by increased intraocular pressure (IOP) and retinal ganglion cell (RGC) death. Lowering IOP remains the only proven intervention for all forms of glaucoma (1, 2). IOP is primarily regulated by the balance between aqueous humor production in the ciliary body and its outflow in the trabecular meshwork. Medications targeting the ciliary body to lower IOP have systemic complications, including bradycardia, metabolic acidosis, and kidney stone formation (3, 4). Additionally, low patient adherence to long-term self-administration of IOP-reducing eye drops is a significant challenge that contributes to glaucoma progression (5, 6). Because glaucoma is a severe and chronic condition requiring lifelong treatment, a targeted therapy that is precise and long-lasting is needed to optimize medical management.

Carbonic anhydrase type 2 (CA2) in the nonpigmented ciliary epithelium is well-recognized for its role in aqueous humor secretion by affecting bicarbonate transport (7). Systemic carbonic anhydrase inhibitors (CAI) such as acetazolamide and topical CAI agents such as dorzolamide are widely used to treat glaucoma (8); however, chronic treatment of systemic CAI can lead to complications such as kidney stone formation. Another transmembrane protein, aquaporin 1 (AQP1), permits passive transport of water along an osmotic gradient. It is expressed in ciliary epithelial cells and has been shown to contribute to aqueous humor secretion (9, 10). We hypothesize that disruption of these genes in the ciliary body will block water transport and reduce aqueous humor production.

CRISPR/Cas technology has emerged as a revolutionary gene manipulation technique. Cas13, a family of RNA-targeting CRISPR effectors, has demonstrated the ability to efficiently and precisely edit cellular RNAs in mammalian cells in vitro and in vivo (11–13). It is more precise and efficient than other RNA interference methods. Unlike the genomic DNA-targeting CRISPR–Cas9 system, Cas13 targets RNA for therapeutic applications, thereby eliminating the risk of introducing permanent changes to the genome. hfCas13X and hfCas13d are two high-fidelity Cas13 variants that induce efficient on-target activity with minimal transcriptome-wide off-target effects (14).

In this study, we compare the editing efficiency of the two Cas13 variants and show that the better-performing hfCas13d lowers IOP by disrupting two transmembrane proteins, AQP1 and CA2, in the mouse ciliary body. Cas13d treated mice show significantly lower IOP than untreated control groups in both wild-type and glaucoma mouse models, whereas functional measures of RGCs (P1–N2 amplitude by pattern electroretinogram [PERG]), retina thickness and ciliary morphology do not differ among the groups. Additional studies show that the mRNA and protein expression of *AQP1* and *CA2* decreases after hfCas13d-mediated RNA editing. These data together indicate that Cas13d-mediated gene editing presents a promising therapeutic approach for treating glaucoma.

## Results

### hfCas13d outperforms hfCas13X in RNA knockdown activity in HEK293T, Neuro2a (N2a) and NIH3T3 (3T3) cells

**Fig. 1.**
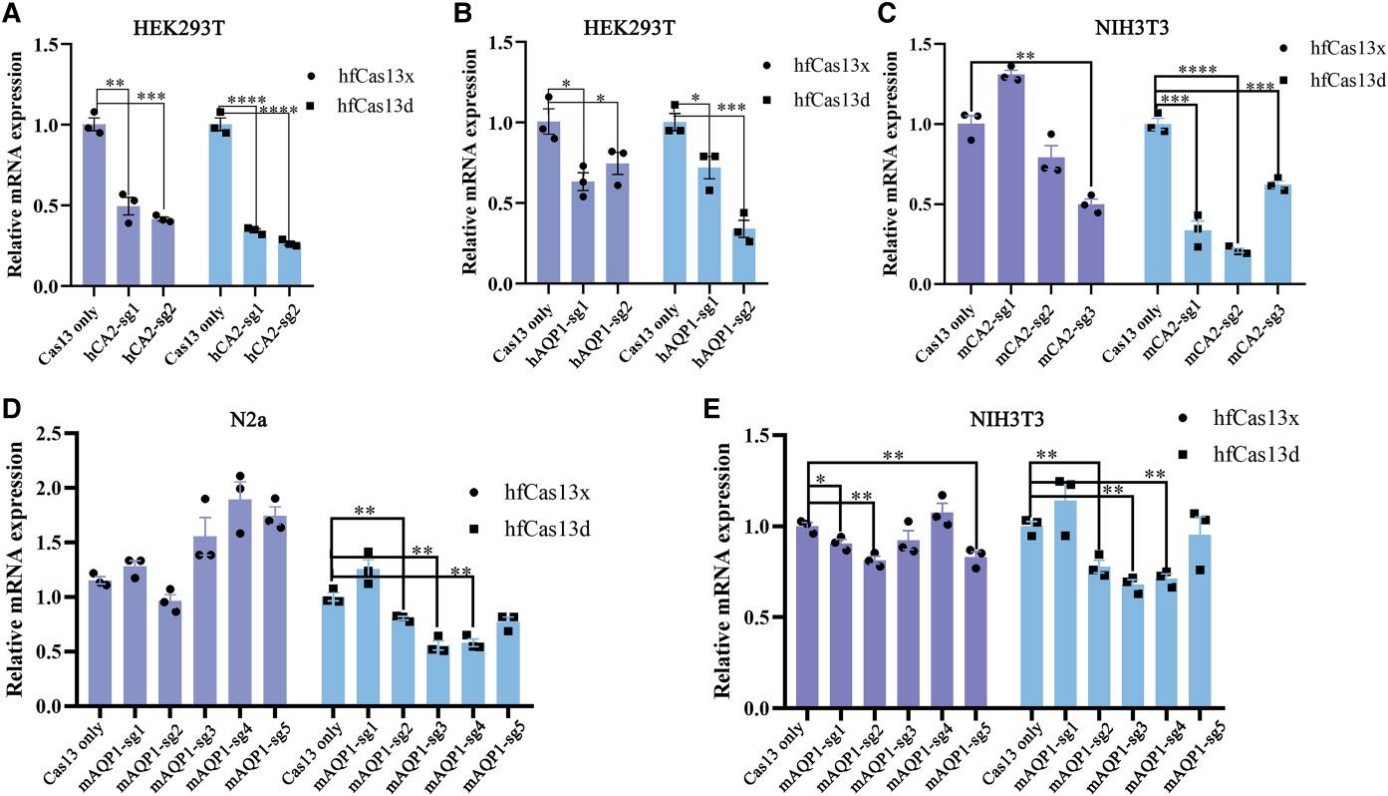
hfCas13d generally outperforms hfCas13x in HEK293T, NIH3T3, and Neuro2a(N2a) cells. A) hfCas13d outperforms hfCas13x in HEK293T cells at two sites targeting CA2. B) hfCas13d outperforms hfCas13x in HEK293T cells at two sites targeting AQP1. C) hfCas13d outperforms hfCas13x in 3T3 cells at three sites targeting CA2. D and E) hfCas13d outperforms hfCas13x in N2a and 3T3 cells at five sites targeting AQP1. Data were analyzed by Student's t test. *P < 0.05, **P < 0.01, ***P < 0.001, and ****P < 0.0001.

Recently, hfCas13X and hfCase13d have been reported to mediate mRNA editing in mammalian cells. To choose an optimal Cas13 system for therapeutic purposes, we compared RNA editing efficiency of hfCas13X and hfCas13d in HEK293T cells. Two gRNAs targeting *CA2* or *AQP1* genes in HEK293T cells were designed and designated as hCA2-sg1, hCA2-sg2, hAQP1-sg1, and hAQP1-sg2, respectively. HEK293T cells were transiently transfected with plasmids encoding hfCas13X or hfCas13d and the corresponding gRNAs as experimental groups, and control groups were transfected with only hfCas13X or hfCas13d (Fig. S1A, Table S1). RNA extracted and assayed by qPCR 48 h posttransfection showed comparable RNA degradation at two CA2 sites by both hfCas systems. Compared to cells treated only with hfCas13X or hfCas13d, the CA2 mRNA levels decreased by 58.7 ± 4.1% in cells treated with hfCas13X and gRNAs and by 73.7 ± 4.1% in cells treated with hfCas13d and gRNAs (Fig. 1A). Similarly, both hfCas13 systems significantly disrupted RNA expression at two AQP1 sites, but hfCas13d outperformed hfCas13X at hAQP1-sg2: a decrease in AQP1 mRNA level of 66.3 ± 7.5% compared to 37.3 ± 9.6% (Fig. 1B).

After the pilot study, we screened gRNAs in mouse cell lines for gene delivery. We first examined if *CA2* and *AQP1* genes are expressed in mouse cell lines. RT-PCR showed that *AQP1* is expressed in both NIH3T3 and N2a cell lines, but *CA2* is only expressed in NIH3T3 cells (Fig. S1B). We next designed and tested a panel of three gRNAs targeting *CA2* (mCA2-sg1 to mCA2-sg3) and five gRNAs targeting *AQP1* (mAQP1-sg1 to mAQP1-sg5). The qPCR results showed that hfCas13d outperformed hfCas13X in mouse cell lines. hfCas13X exhibited a significant decrease in CA2 mRNA level (50.3 ± 6.0%) at one of three target sites (mCA2-sg3) in NIH3T3 cells. In comparison, hfCas13d induced significant CA2 mRNA knockdown at all three sites. The most efficient site was mCA2-sg2, where the mRNA level decreased by 78.9 ± 3.8% (Fig. 1C). Only hfCas13d effectively knocked down AQP1 mRNA in N2a cells: the levels of AQP1 mRNA decreased significantly, ranging from 19.6 ± 4.1% at mAQP1-sg2 to 44.5 ± 5.7% at mAQP1-sg3 (Fig. 1D). In NIH3T3 cells, both hfCas13X and hfCas13d exhibited effective AQP1 RNA knockdown at most sites: AQP1 mRNA knockdown levels induced by hfCas13X ranged from 9.4 ± 2.9% to 18.6 ± 2.9%; with hfCas13d the range was from 22.2 ± 4.4% to 32.0 ± 3.7% (Fig. 1E). Based on these results, we concluded that gene editing of mCA2-sg2 and mAQP1-sg3 in mouse-derived cell lines was more effective by hfCas13d than by hfCas13X.

### Simultaneous knockdown of AQP1 and CA2 by hfCas13d lowers IOP in wild-type mice

**Fig. 2.**
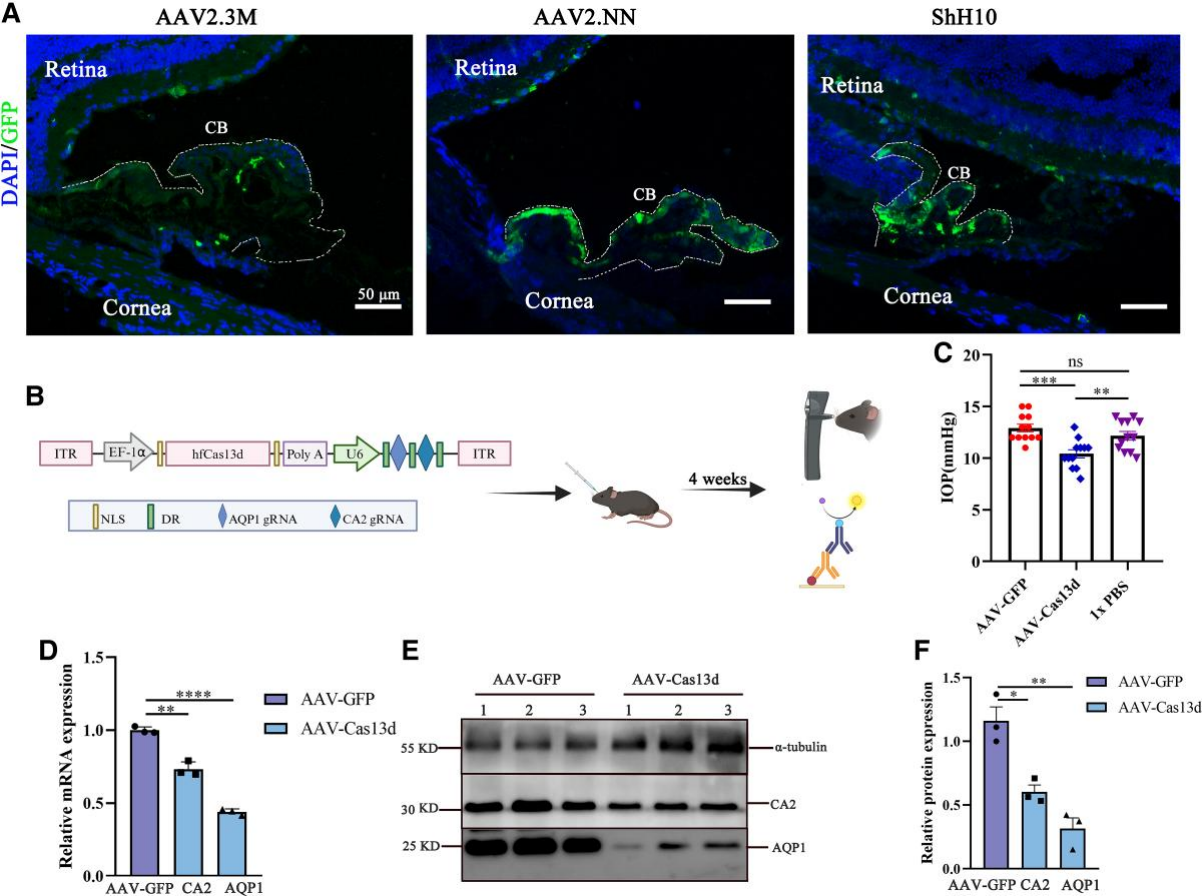
hfCas13d-mediated knockdown of CA2 and AQP1 lowers IOP in wild-type mice. A) A total of 2 × 10^10^ genome copies of different AAV serotypes encoding GFP were injected into the vitreous cavity of one eye of each mouse. B) Diagram of AAV-Cas13d vector and AAV injection. C) IOP of AAV-Cas13d group was reduced after AQP1 and CA2 disruption by a mean of 2.5 mmHg and 1.7 mmHg compared to AAV-GFP and 1× PBS groups, respectively. Paired t test, *n* = 12 pairs. D) qPCR shows decreased CA2 and AQP1 mRNA expression after AAV-Cas13d injection (*n* = 3). E) Representative Western blot image showing decreased CA2 and AQP1 expression. F) Quantification of Western blot images using ImageJ. Data were analyzed by Student's t test. *P < 0.05, **P < 0.01, ***P < 0.001, and ****P < 0.0001.

To select the most efficient AAV serotype for gene editing in the ciliary body, we compared the transduction efficiency of three AAV variants: ShH10, AAV2.3M, and AAV2.NN. ShH10 serotype, an AAV6 variant (I319V, N451D, D532N), has higher transduction efficiency than AAV2/6 and efficiently transduces the ciliary body (15, 16). AAV2.3M is an AAV2 variant with triple mutations (Y444,500,730F). AAV2.NN, another AAV2 variant with a peptide insertion, has been shown to have increased transduction efficiency in the retina compared to the wild-type AAV2 (17, 18). These three AAV serotypes encoding GFP driven by CAG promoter were intravitreally injected into 8-week-old wild-type C57BL/6J mice. ShH10 serotype demonstrated the highest GFP signal in ciliary body 4 weeks after injection, followed by AAV2.NN; AAV2.3M exhibited only weak GFP signal in ciliary body (Fig. 2A). Based on its high transduction efficiency in the ciliary body, we used ShH10 serotype for further study. mCA2-sg2 and mAQP1-sg3 were cloned into the same plasmids encoding hfCas13d to generate ShH10-hfCas13d-AQP1-CA2 (hereafter referred to as AAV-Cas13d) (Fig. 2B). These viral particles were generated and used for gene delivery in vivo.

To assess the delivery efficacy, wild-type C57BL/6J mice were intravitreally injected with AAV-Cas13d or ShH10-CAG-GFP (hereafter referred to as AAV-GFP), and the contralateral eye was injected with 1× PBS as control. Four weeks postinjection, IOP decreased by a mean of 2.5 ± 0.5 and 1.750 ± 0.6 mmHg in the AAV-Cas13d treated group compared to the AAV-GFP and 1× PBS groups, respectively (Fig. 2C). This represents a 19.4 and 14.0% reduction in IOP compared to the AAV-GFP- and 1× PBS-treated controls, respectively. CA2 and AQP1 expression levels in the ciliary body were identified by qPCR and western blotting assays. The results showed a 26.7 ± 3.2% decrease in CA2 mRNA levels and a 56.1 ± 1.8% decrease in AQP1 mRNA levels (Fig. 2D). Western blotting results confirmed the knockdown of CA2 and AQP1, showing a significant reduction in CA2 and AQP1 protein levels in AAV-Cas13d treated mice as compared to the AAV-GFP-treated groups (Fig. 2E and F). These results indicate that AAV-Cas13d simultaneously knocks down CA2 and AQP1 in the mouse ciliary body.

### Intraocular delivery of AAV-Cas13d does not cause adverse structural or functional effects in mouse retina

To assess any possible adverse effects of AAV-Cas13d treatment, wild-type mice treated with AAV-Cas13d or AAV-GFP, or untreated controls were subjected to spectral-domain optical coherence tomography (SD-OCT) imaging 4 weeks postinjection. Retina thickness and retinal ganglion cell complex (GCC) thickness are well-accepted indicators of retinal health (19–21); we measured the retina thickness and GCC thickness based on the OCT images of the mouse retina area surrounding the optic nerve head (Supplementary Fig. S2A). The results exhibited no differences in GCC and retina thickness between the treatment and control groups (Supplementary Fig. S2B and C).

To assess the function of retinas treated with AAV-Cas13d, we used PERG, a highly sensitive electrophysiological assay, to assess retinal ganglion cell function (22). AAV-Cas13d and AAV-GFP-treated eyes examined by PERG revealed no significant differences in the peak-to-trough (P1–N2) amplitude ratio (Supplementary Fig. S3A and B). Histological examination also showed no structural abnormalities in the ciliary body (Supplementary Fig. S3C). These results demonstrate that AAV-Cas13d treatment does not change retina structure or function or ciliary body morphology.

### Simultaneous knockdown of AQP1 and CA2 by hfCas13d lowers IOP in a dexamethasone-induced ocular hypertension model

**Fig. 3.**
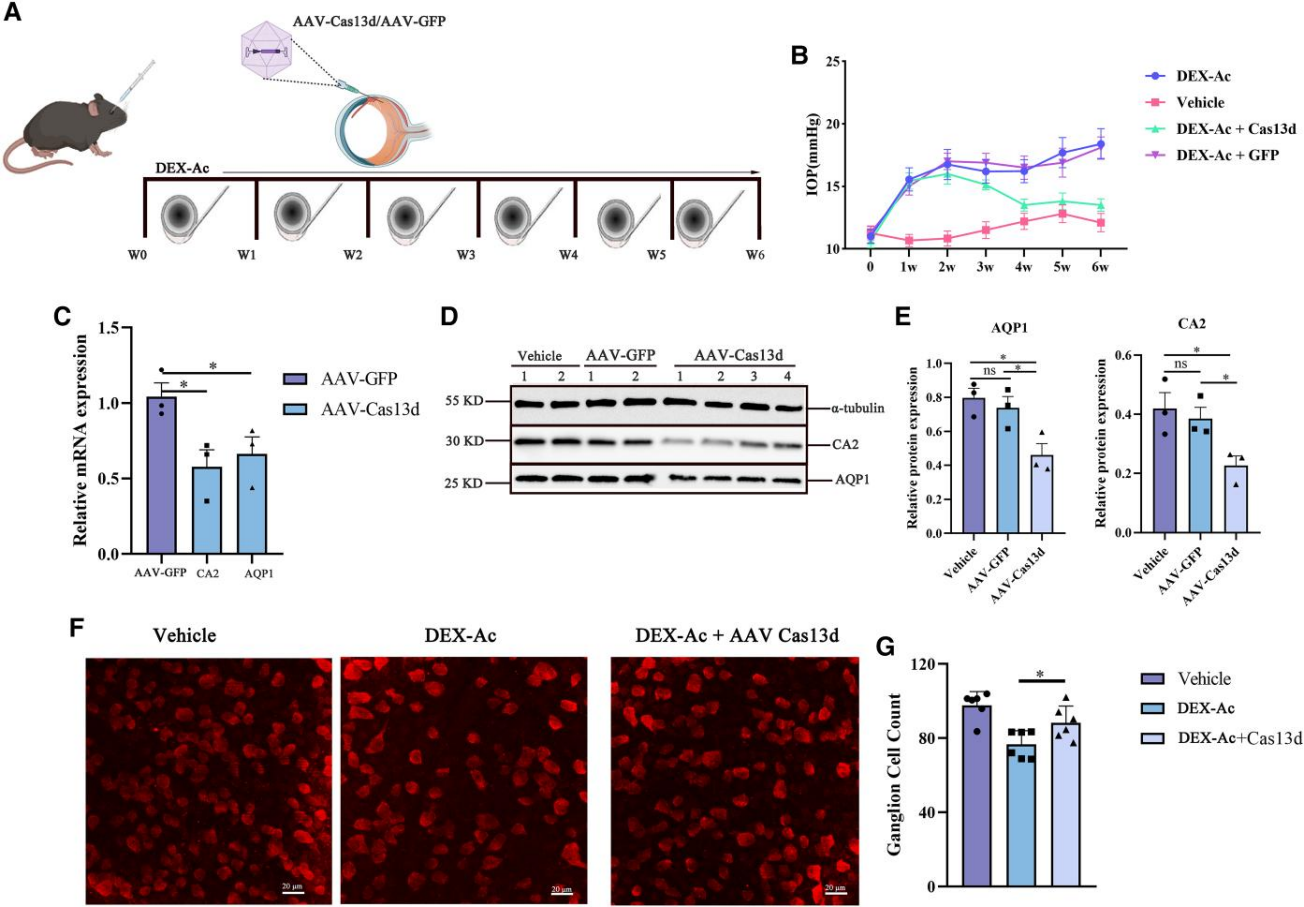
hfCas13d-mediated disruption of CA2 and AQP1 lowers IOP in mouse models of ocular hypertension. A) Diagram showing generation of dexamethasone-induced glaucoma model and AAV-Cas13d injection. DEX-Ac, dexamethasone 21-acetate. Dex-Ac was administrated twice weekly for 6 weeks. 2× 10^10^ genome copies of AAV-GFP or AAV-Cas13d were administered in the second week after DEX-Ac treatment. B) AAV-Cas13d treatment changes IOP by a mean of 3.7 ± 1.1 mm Hg. *n* = 10 in each group. DEX-Ac significantly increased IOP 2 weeks after treatment compared with the vehicle-treated group. AAV-Cas13d treatment prevents IOP from further increasing compared with the AAV-GFP-treated group. C) qPCR shows decreased CA2 and AQP1 mRNA expression after AAV-Cas13d treatment. D and E) Representative Western blot image showing decreased CA2 and AQP1 expression after AAV-Cas13d treatment. Data were analyzed by Student's t test. *P < 0.05, **P < 0.01, ***P < 0.001. F) Representative images of RGC quantification after AAV-Cas13d treatment. G) Counts of RGCs stained by RBPMS in retinal flat mounts after treatment with vehicle, DEX-Ac or DEX-Ac + AAV-Cas13d. Mean of 12 236.2 μm × 236.2 μm fields per eye were quantified as the mean ± SD across three independent experiments; unpaired Student's t test, *n* = 6. Scale bars, 20 μm. *P < 0.05.

AAV-Cas13d-mediated AQP1 and CA2 knockdown was tested in a dexamethasone-induced ocular hypertension model. Dexamethasone 21-acetate (DEX-Ac) was administered for 6 weeks to both eyes of 6- to 8-week-old mice by periocular conjunctival fornix injection as reported previously (23). Vehicle-treated mice were used as control. DEX-Ac-treated mice received an intravitreous injection of AAV-Cas13d or AAV-GFP at the second week (Fig. 3A). IOP was measured in mice treated with DEX-Ac, vehicle, DEX-Ac combined with AAV-Cas13d, or DEX-Ac combined with AAV-GFP. Four weeks after injection, IOP was dramatically increased in mice treated with DEX-Ac or DEX-Ac combined with AAV-GFP compared with those receiving vehicle alone (Fig. 3B). In mice receiving DEX-Ac combined with AAV-Cas13d, IOP was higher than in mice receiving vehicle alone, but markedly lower than in those treated with DEX-Ac only (Fig. 3B). AAV-Cas13d treatment reduced IOP by an average of 3.7 ± 1.1 mm Hg (21.8% reduction) compared to mice receiving DEX-Ac and prevented further elevation. qPCR showed that AAV-Cas13d induced a significant decrease in CA2 and AQP1 mRNA levels (Fig. 3C), and western blotting demonstrated significantly decreased CA2 and AQP1 expression (Fig. 3D and E). Because RGC death is a hallmark of glaucoma, we examined RGC survival by immunostaining against RNA binding protein (RBPMS) 4 weeks after AAV-Cas13d treatment (Fig. 3F, Supplementary S4A). Retina wholemounts showed that AAV-Cas13d treatment increased RGC survival in the peripheral retina (Fig. 3G), which provides evidence supporting this approach as a novel treatment for glaucoma.

We subsequently performed PERG to evaluate RGC function. However, AAV-Cas13d-, vehicle- and AAV-GFP-treated eyes examined by PERG revealed no significant differences in the peak-to-trough (P1–N2) amplitude ratio (Supplementary Fig. S5A−D).

### AAV-Cas13d-mediated knockdown of AQP1 and CA2 does not change outflow facility in DEX-Ac-induced ocular hypertension model

**Fig. 4.**
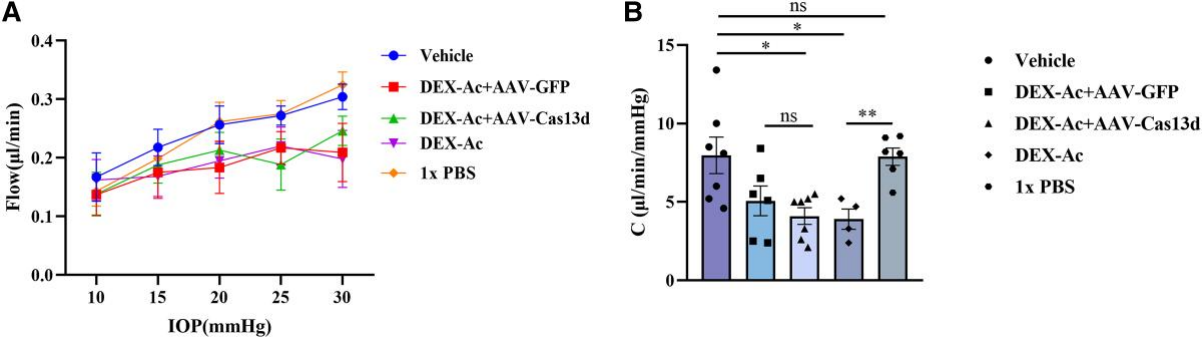
AAV-Cas13d treatment does not change outflow facility. A) Perfusion plots of mouse eyes injected with vehicle, DEX-Ac + AAV-Cas13d, DEX-Ac + AAV-GFP, DEX-Ac only, or 1× PBS. B) No difference in outflow facility in eyes treated with DEX-Ac + AAV-Cas13d, DEX-Ac + AAV-GFP, or DEX-Ac only; significantly lower outflow facility in eyes treated with DEX-Ac than treated with vehicle or 1× PBS. (*n* = 7 in vehicle-treated group; *n* ≥ 6 eyes in AAV-treated groups, unpaired Student's t test).

To determine whether AAV-Cas13d treatment affects outflow facility, we performed perfusion analysis. The eyes of the dexamethasone-treated groups (DEX-Ac + AAV-Cas13d, DEX-Ac + AAV-GFP, and DEX-Ac) demonstrated a significant decrease in average outflow facility (4.1, 5.1, and 3.9 nL/min/mmHg, respectively) compared with eyes receiving vehicle or 1× PBS (8 and 7.9 nL/min/mmHg) (Fig. 4A and B). Notably, outflow facility in the groups receiving DEX-Ac+AAV-Cas13d or DEX-Ac+AAV-GFP did not differ. Similarly, groups receiving vehicle or 1× PBS showed no difference (Fig. 4B). These data showed that DEX-Ac treatment lowered outflow facility, whereas AAV-Cas13d treatment did not affect outflow facility, implying that the change in IOP is due to reduced AQP1 and CA2 expression.

## Discussion

We demonstrated a novel approach to treating ocular hypertension and glaucoma by targeting mRNA gene expression with CRISPR–Cas. Our goal was to use a Cas13 RNA editing system to knock down two genes associated with aqueous humor production, *Aqp1* and carbonic anhydrase (*CA2*). CAIs, which suppress aqueous humor production, have been commonly used for decades in the management of glaucoma (24–27). Aquaporin-1 (AQP1) is a water-transporting protein which has been implicated in aqueous humor production (28).

The present study demonstrates that simultaneous knockdown of AQP1 and CA2 in the ciliary body lowers IOP in wild-type mice and a steroid-induced ocular hypertension mouse model. During the preparation of our manuscript, another group published a study that pointed out *CA2* knockout significantly and sustainably reduces IOP for up to 2 months in normal mice and glaucoma models by inhibiting aqueous humor production (29). These investigators used a dual-AAV strategy to deliver CRISPR/Cas9 components: one AAV vector packaged two sgRNAs targeting *CA2*; the other AAV delivered miniCMV-SpCas9.

The hfCas13d-mediated RNA editing that we used offers several advantages. First, it circumvents patient adherence issues requiring daily eye drop administration. Second, it enables RNA degradation without permanently changing genomic DNA. Third, the gene editing events induced by hfCas13d are reversible, allowing treatment to be flexibly adjusted to accommodate the needs of the individual patient. For example, the dose and frequency of AAV-Cas13d can be increased for patients in late stages of disease, and the dose and/or frequency decreased as IOP responds. Similarly, patients with milder diseases can begin with lower doses, which can be adjusted as necessary. In this respect, RNA editing is superior to DNA editing, which is irreversible. In addition, hfCas13d is a high-fidelity gene editing system that exhibits on-target activity with efficiency comparable to that of wild-type Cas13d while inducing little to no transcriptome-wide off-target or cell growth effects. These attributes make hfCas13d a highly advantageous system for therapeutic application.

Future research will determine the strategy to optimize hfCas13d-mediated gene therapy for individual patients, including the timing and dosage and optimal AAV serotypes. For example, hfCas13d allows for adjustable gene silencing with repetitive applications, and inducible promoters or dosage-controlled delivery systems may also be used to adjust the effects of AAV-Cas13d treatment noninvasively. In addition, Modifying the administration method and developing an optimal AAV serotype that avoids penetration into peripheral tissues are necessary for therapeutic purposes. Unwanted transduction in peripheral tissues may affect safety. The ShH10 used in this study transduces mouse retina after intravitreous injection. It would affect retinal health. To address the issue, we can either evolve an AAV serotype that precisely transduces nonpigmented epithelial cells in the ciliary body or perform subconjunctival injections to restrict AAV spreading locally. Future studies should also treat glaucoma models of varying severity with different AAV doses to match treatment dosage with the severity stage of the disease.

## Materials and methods

### Plasmids construction

The hfCas13d (#190034), hfCas13X (#190033), and AAV-hfCas13d (#191795) plasmids were obtained from Addgene. The gRNAs were designed by Cas13 design tools (30, 31). The hfCas13d-gRNAs or hfCas13X-gRNAs were generated by T4 ligation of annealed oligos into the Bpi*I*-digested hfCas13d or hfCas13X plasmid. The AAV-hfCas13d-AQP1-CA2 plasmid was constructed through subsequent digestion and ligation using the Nhe*I* and EcoR*I* enzymes. The sequences of gRNAs are listed in Supplementary Table S1.

### Cell culture and DNA transfection

Cell culture and DNA transfection were carried out following the procedures outlined in our previous study (32). Briefly, HEK293T (Life Technologies), neuro2a (N2a), and NIH3T3 (3T3) cells were cultured in Dulbecco's modified Eagle's medium (10013CV; Corning) supplemented with 10% FBS (F7524; Sigma-Aldrich), 1 mM GlutaMAX (Life Technologies), 100 U/mL penicillin, and 100 mg/mL streptomycin, and incubated at 37 °C with 5% CO_2_. The cells were seeded onto 12-well poly-D-lysine-coated plates (Corning Life Sciences). Approximately 12–15 h after plating, cells were transfected with 2.25 µL of PolyJet In Vitro DNA Transfection Reagent (SignaGen, Catalog #: SL100688) using 375 ng of hfCas13d or hfCas13x plasmids and 375 ng of gRNA plasmids.

### Animals

As mentioned in our previous study (33), all in vivo experiments were conducted in accordance with the guidelines set by the Association for Research in Vision and Ophthalmology Statement for the Use of Animals in Ophthalmic and Vision Research and were approved by the Institutional Animal Care and Use Committee at Stanford University School of Medicine. Wild-type (C57BL/6J) mice were sourced from The Jackson Laboratories (Bar Harbor, ME). To minimize age-related variability, all mice were within the same age range. The mice were housed under a 12-h light/dark cycle with free access to water and food. Ketamine injections were administered based on the mice's body weight (100 mg/kg). Oxygen flow was set to 2 L/min; isoflurane was 1% and delivered by nose cone.

### Quantitative real-time PCR

qPCR was performed following the procedures outlined in our previous study (32). Briefly, total RNA was extracted 48 h after transfection using TRIzol reagent (Invitrogen, Catalog no. 15596026). cDNA was synthesized using the HiScript II 1st Strand cDNA Synthesis Kit (Vazyme, Cat. no. R212). Primers for quantitative real-time PCR are provided in Table S2. Quantitative real-time PCR was carried out with the BioEasy SYBR Green I real-time PCR kit on the Bio-Rad CFX Opus 384 multicolor real-time PCR detection system. Relative gene expression, normalized to Gapdh, was calculated using the 2-ΔΔCT method. The normalized data of control groups and the experimental groups are averaged in each experiment, and the experiment is repeated three times. All gene expression data were derived from experiments performed in triplicate, and the results are presented as mean ± SEM.

### Western blotting analysis

For Western blotting, the ciliary bodies of both experimental and control mice were dissected and homogenized in 60 μL of RIPA Lysis Buffer (Millipore Sigma, Cat. No. 20-188). Protein concentrations were determined using the PIERCE BCA Protein Assay Kit (Thermo Scientific, Cat. no. 23227). A total of 15 μg of protein from each sample was loaded. To assess changes in AQP1 expression, recombinant antiaquaporin 1 antibody (1:800; Abcam, ab300463) and anti-α-tubulin polyclonal antibody (1:5,000; Proteintech, Cat. no. 11224-1-AP) were used as primary and internal control antibodies, respectively. The polyvinylidene fluoride (PVDF) membrane was then stripped using western blot stripping buffer (Santa Cruz Biotechnology, sc-281698) before CA2 polyclonal antibody (1:1,000; Proteintech, 16961-1-AP) was applied. Signals were acquired by directly measuring chemiluminescence using a digital camera (Amersham Imager 600), and quantification was performed with ImageJ software from at least three independent experiments. The relative CA2 and AQP1 expression levels of the control groups and the experimental groups normalized to α-tubulin are averaged in each experiment, and the experiment is repeated three times.

### Dexamethasone-induced ocular hypertension

Dexamethasone-induced ocular hypertension model was generated as reported in a previous study (23). Briefly, 10 mg/mL of DEX-Ac suspension was prepared by mixing 0.1 g dexamethasone 21-acetate (AK Scientific; CAS# 1177-87-3) with 10 mL vehicle suspension. Four to 6-week-old C57BL/6J mice received a DEX-Ac suspension or vehicle suspension (20 µL) by conjunctival injection over a period of about 20 seconds. Each animal received a subconjunctival injection in both eyes of either DEX-Ac suspension or vehicle suspension. The injection was performed once a week with a 32-gauge needle and a glass micro-syringe (25 mL volume; Hamilton Company).

### AAV production

ShH10-hfCas13-AQP1-CA2 (1 × 10^13^ gc/mL) and ShH10-CAG-GFP (3.74 × 10^13^ gc/mL) were commercially obtained from AAVnergene Inc (Rockville, MD).

### Intravitreal injection

Intravitreal injection was performed following the protocol utilized in our previous study (34). Briefly, mice were anesthetized with a combination of xylazine and ketamine based on their body weight (0.01 mg xylazine/g + 0.08 mg ketamine/g). For ShH10-hfCas13-AQP1-CA2, ShH10-CAG-GFP, vehicle, and 1× PBS intravitreal injections, a pulled and polished microcapillary tube was inserted into the peripheral retina of 4- to 6-week-old mice, just behind the ora serrata. Approximately 2 μL of vitreous humor was removed to facilitate the injection of 2 μL (∼2 × 10^10^ GC) of AAV or control solutions into the vitreous chamber. Both wild-type and DEX-Ac-induced ocular hypertension mice were treated with a final dose of 2 × 10^10^ GC of AAV-Cas13d or AAV-GFP in each eye via intravitreal injection.

### IOP measurements

The IOP measurement was performed between 4 PM and 6 PM PST with the Icare TonoLab tonometer (TV02; Icare Finland Oy, Espoo, Finland). Briefly, mice were anesthetized via nose cone delivery of isoflurane and oxygen. Proparacaine topical anesthesia was applied to each eye, and IOP was measured 1 min later. The average of five readings was recorded as the IOP.

### SD-OCT imaging

OCT imaging was conducted using the OCT mode with a 30° licensed lens (Heidelberg Engineering), following previously described protocols (34, 35). Briefly, the mouse retina was scanned in ring scan mode, centered on the optic nerve head, with 100-frame averaging under high-resolution settings (each B-scan comprising 1536 A-scans). The GCC, comprising the retinal nerve fiber layer, ganglion cell layer, and inner plexiform layer, was analyzed. The average GCC thickness around the optic nerve head was measured manually using Heidelberg software.

### Outflow facility measurements

The mouse eye perfusion apparatus was described previously (33). Eyes were perfused over a 50-min period at incremental IOP levels. The outflow facility of each sample and its relationship with IOP and the corresponding outflow rate were determined using the modified Goldmann equation: *F* = *C* × IOP + *U*, where *C* represents the conventional outflow rate. Flow rates and corresponding IOP values were plotted using GraphPad Prism, and the conventional outflow rate (*C*) for each sample was calculated.

### PERG recording

PERG was performed following protocols utilized in previous studies (34, 36). Briefly, PERG recordings were performed simultaneously for both eyes using the Miami PERG system (Intelligent Hearing Systems, Miami, FL). A feedback-controlled heating pad (TCAT-2LV, Physitemp Instruments Inc., Clifton, NJ) maintained the animal's core temperature at 37 °C. Prior to recording, a small amount of lubricant eye drop (Systane) was applied to prevent corneal opacities. The reference electrode was placed subcutaneously on the back of the head between the ears, the ground electrode at the root of the tail, and the active steel needle electrode subcutaneously on the snout for simultaneous acquisition of responses from both eyes. Two 14 × 14 cm LED-based stimulators were positioned 10 cm from each eye, centered in front of the animal. The stimulus pattern featured a contrast of 85% and a luminance of 800 cd/m², consisting of four cycles of black–gray elements with a spatial frequency of 0.052 cycles/degree. Independent PERG signals were recorded asynchronously for both eyes, with each trace capturing up to 1020 ms. For each readout, two consecutive recordings of 200 traces were averaged. The first positive peak (P1) in the waveform was observed at ∼100 ms, and the second negative peak (N2) followed. The P1–N2 amplitude was measured, and the mean amplitude from the eye receiving treatments (AAV-Cas13d, AAV-GFP, or DEX-Ac) was compared to the contralateral control eye (1× PBS, vehicle) to calculate the percentage change in amplitude. All amplitude measurements were performed by investigators blinded to the treatment conditions.

### Histology

Histology was performed as described previously (34). Briefly, mouse eyes were collected and lenses removed before fixation in 4% paraformaldehyde. The eyeballs were sequentially dehydrated in 15 and 30% sucrose solutions overnight before being embedded in Tissue-Plus OCT compound on dry ice. Serial cross-sections (15 μm thick) were prepared using a Leica cryostat, collected on Superfrost Plus Slides, and stored at −80 °C until further processing. Prior to hematoxylin and eosin staining, the slides were baked at 37 °C for up to 2 h. Images were captured using a Keyence BZ-X800 Microscope.

### Immunohistochemistry of retinal RGC counts

RGCs count was performed as in a previous study (37). Briefly, after mice were anesthetized and perfused through the heart with 4% PFA in PBS, the eyes were removed and postfixed with 4% PFA overnight at 4 °C. The following day, retinas were thoroughly rinsed with 1× PBS for 30 min. This procedure was repeated three times, followed by blocking with blocking buffer (5% BSA + 0.2% tripsin-×100 in 1× PBS) for 2 h at room temperature. Floating retinas were incubated overnight at 4 °C with RBPMS guinea pig antibody (ProSci, California) and subsequently washed three times with 1× PBS for 30 min each. The retinas were then incubated with Alexa Fluor 647-goat antiguinea pig antibody for 1 h at room temperature, followed by another series of three 30-min washes with PBS. Finally, a cover slip was mounted using Fluoromount-G (SouthernBiotech, Birmingham, Alabama). For RGC counting, 12 fields of 236.2 μm × 236.2 μm area were sampled on average from peripheral regions of each whole retina using a Zeiss LSM 880 microscope with a 20× objective lens (Zoom 1.8), and RBPMS^+^ RGCs were counted by ImageJ (NIH).

### Statistical analysis

All data are presented as mean ± SEM from at least three independent determinations for all experiments. Statistical analyses were performed using Student's t test in GraphPad Prism software version 8.0.1. A *P*-value of <0.05 (*P* < 0.05) was considered statistically significant, with significance levels denoted as *P* < 0.05 (*), *P*  *<* 0.01 (**), *P*  *<* 0.001 (***), and *P* < 0.0001 (****).

## Supplementary Material

pgaf168_Supplementary_Data

## Data Availability

All data produced or analyzed in this study are provided within this published article and its Supplementary materials.
