# Supplementary material for: Gene therapy for ocular hypertension using hfCas13d-mediated mRNA targeting
Source: PNAS Nexus. 2025 Jun 17;4(6):pgaf168. doi: 10.1093/pnasnexus/pgaf168 (PMC12199245; doi:10.1093/pnasnexus/pgaf168)
Supplement: pgaf168_Supplementary_Data [file pgaf168_supplementary_data.docx]

**Gene Therapy for Ocular Hypertension using hfCas13d-mediated mRNA Targeting**

Siyu Chen^1#^, Zhiquan Liu^1#^, Chien-Hui Lo^1^, Qing Wang^1^, Ke Ning^1^, Qi Zhang^1^, Jingyu Zhao^1^, Yingchun Shen^3^, Yang Sun^1,2,*^

1 Department of Ophthalmology, Stanford University School of Medicine, 1651 Page Mill Road, Rm 2220, Palo Alto, CA 94304, USA.

2 Palo Alto Veterans Administration, Palo Alto, CA, USA

3 John A. Burns School of Medicine, 651 Ilalo St, Honolulu, HI 96813, USA

*To whom correspondence should be addressed at: Department of Ophthalmology, Stanford University School of Medicine, 1651 Page Mill Road, Rm 2220, Palo Alto, CA 94304, USA.

Tel: +1 [(650) 724-3952](tel:(650)%20724-3952); Fax: (650) 565-8297; E-mail: yangsun@stanford.edu

# Those authors contribute equally to this study.

**Supplementary Data:**

**
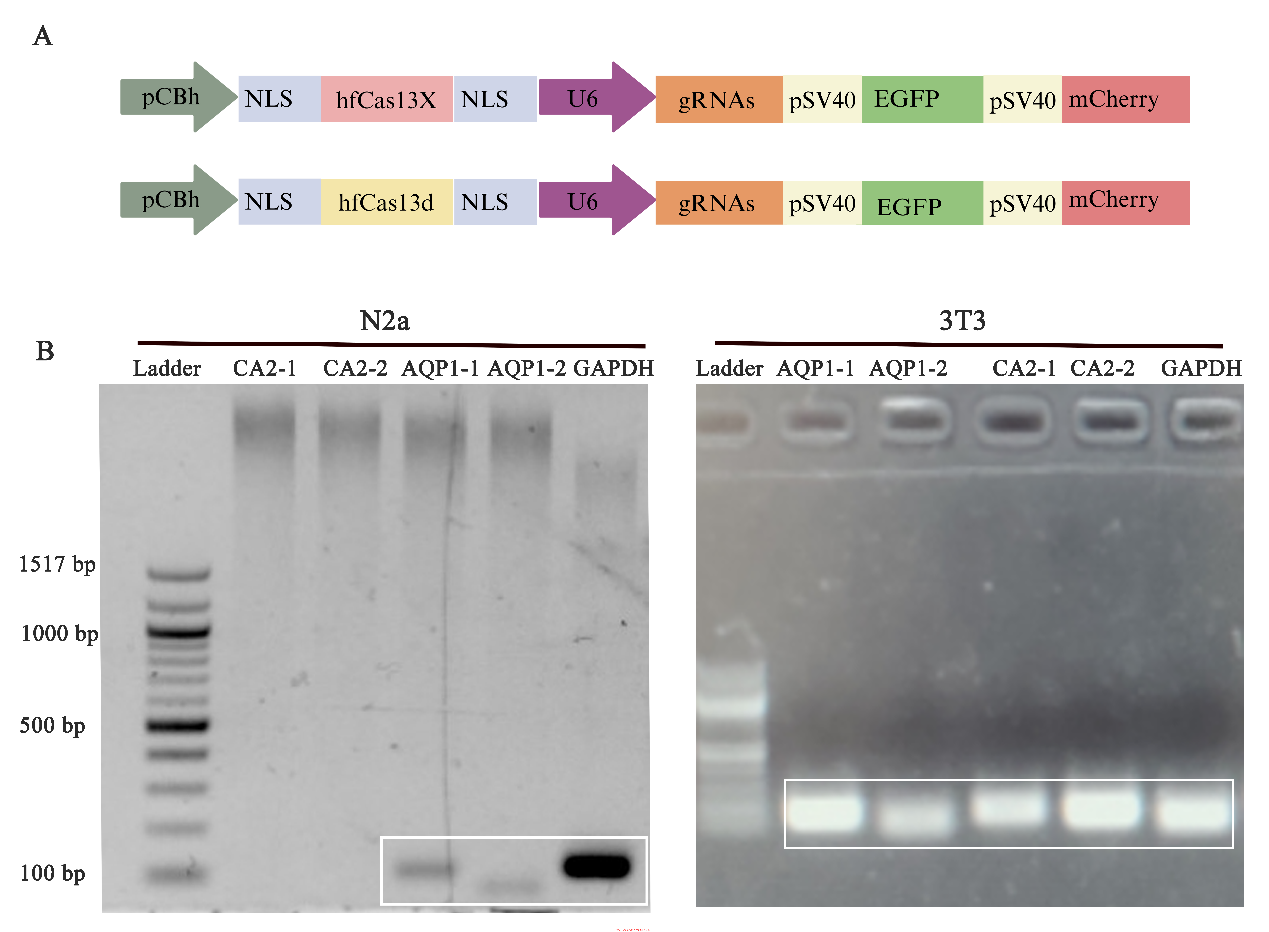
**

**Supplementary Figure 1: Expression profiles of *AQP1* and *CA2* in two mouse cell lines.** (A) Schematic representation of hfCas13d and hfCas13X used to knock down AQP1 and CA2 mRNA. (B) RT-PCR results indicate the expression of *AQP1* in both N2a and 3T3 cells, and the expression of *CA2* in 3T3 cells.

**
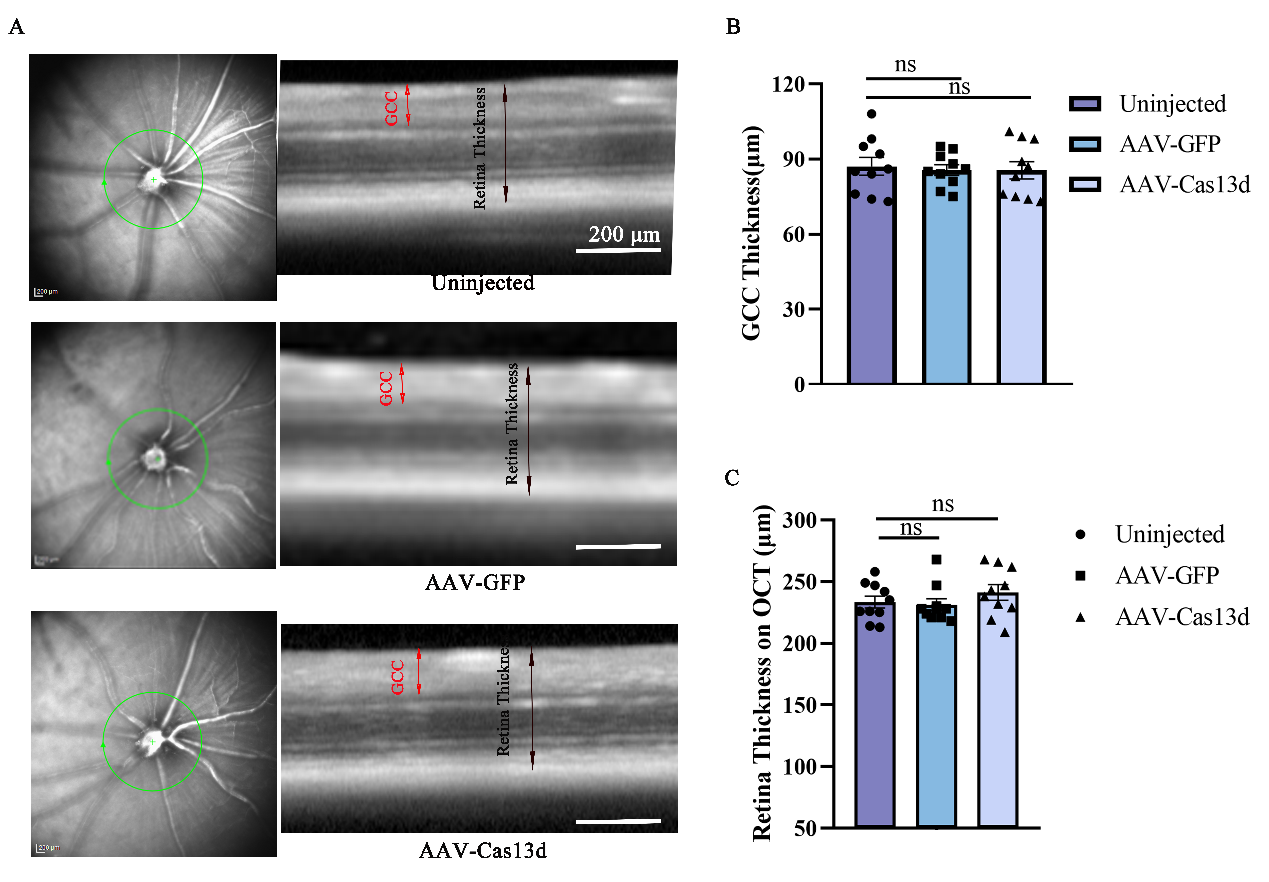
**

**Supplementary Figure 2: The functional impact of AAV-Cas13d treatment on mouse retinas.**

1. Representative OCT images of mouse retinas from wild-type mouse eyes, AAV-Cas13d treated eyes and contralateral control treated with AAV-GFP at 4 weeks post intravitreal injection. GCC: ganglion cell complex, including RNFL, GCL, and IPL layers; indicated as double end arrows. (B) Quantification of GCC thickness in the eyes of wild-type mouse, AAV-Cas13d treated eyes and contralateral control treated with AAV-GFP. (C) Quantification of retina thickness in the eyes of wild-type mouse, AAV-Cas13d treated eyes and contralateral control treated with AAV-GFP. *n*= 10 per group.

**
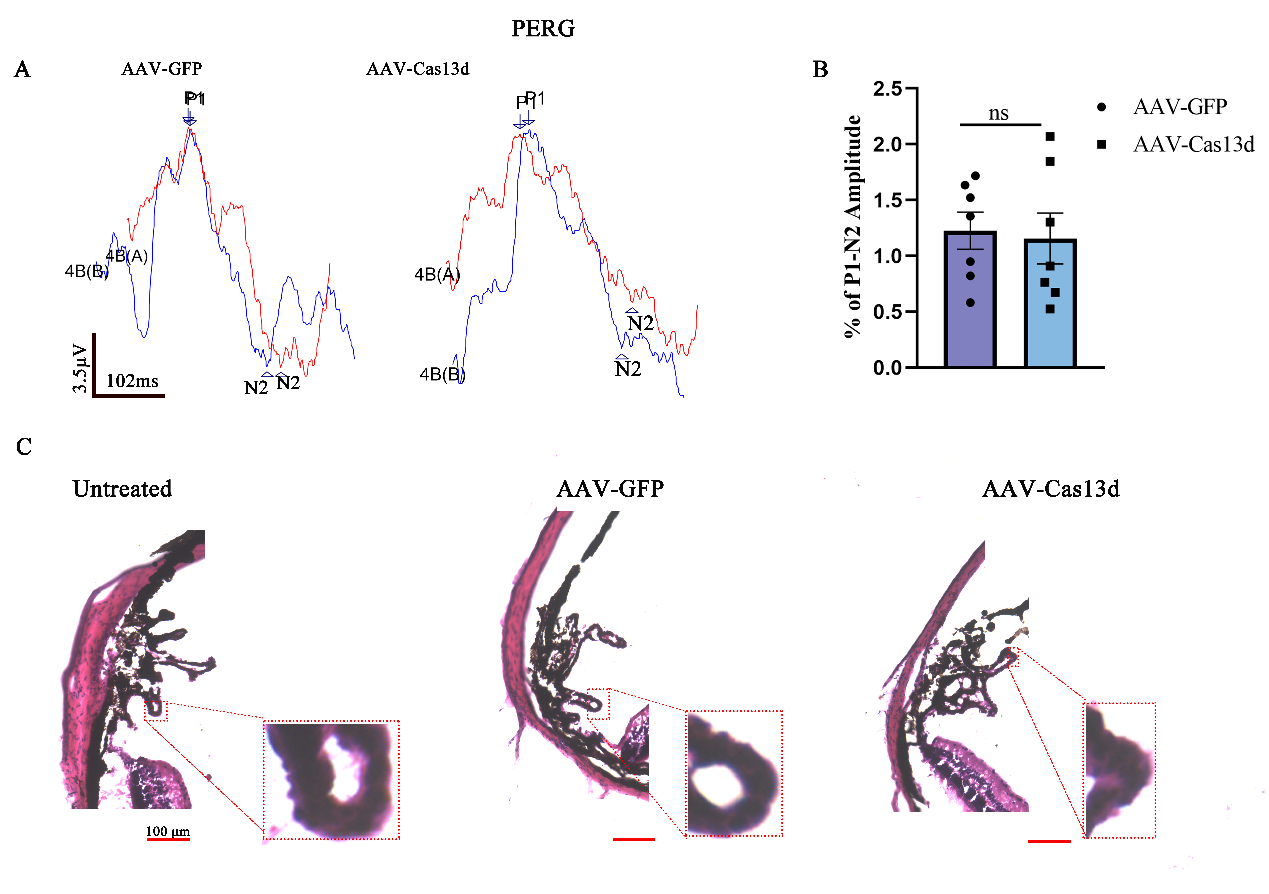
**

**Supplementary Figure 3: The impact of AAV-Cas13d on mouse eyes.** (A) Representative wave forms of PERG at baseline. Blue traces represent 1x PBS treated eyes; Red traces represent AAV-Cas13d/AAV-GFP treated eyes. (B) Quantification of P1-N2 amplitude of PERG four weeks after intravitreal injection. *n*=7 per group (C) Representative H&E staining images of ciliary body of untreated wild-type, AAV-Cas13d and AAV-GFP treated mice. *n*=3 per group, scar bar= 100 μm.


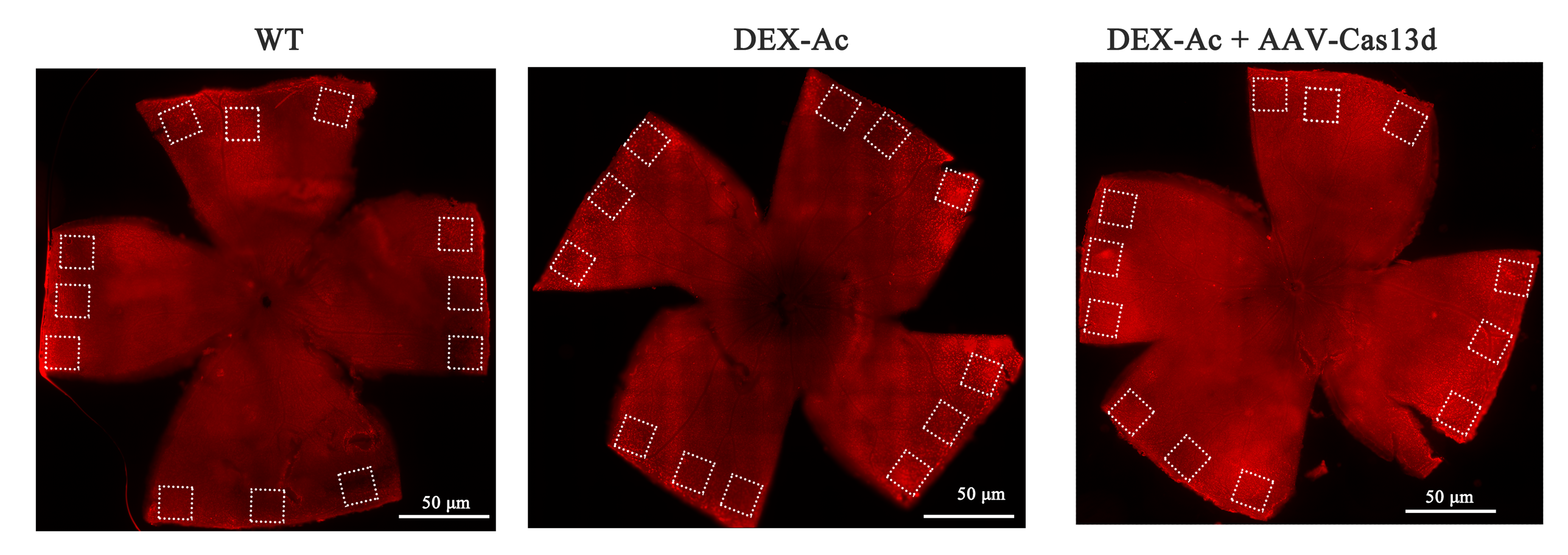


**Supplementary Figure 4: AAV-Cas13d treatment improves peripheral RGC survival in DEX-Ac induced ocular hypertension model.**

1. Representative images of peripheral flat-mounted retinas showing surviving RBPMS-labeled RGCs six weeks after DEX-Ac treatment while four weeks after AAV-Cas13d treatment; scale bar, 50 μm. Dotted rectangles show the area selected in each retina for RGC count analysis.


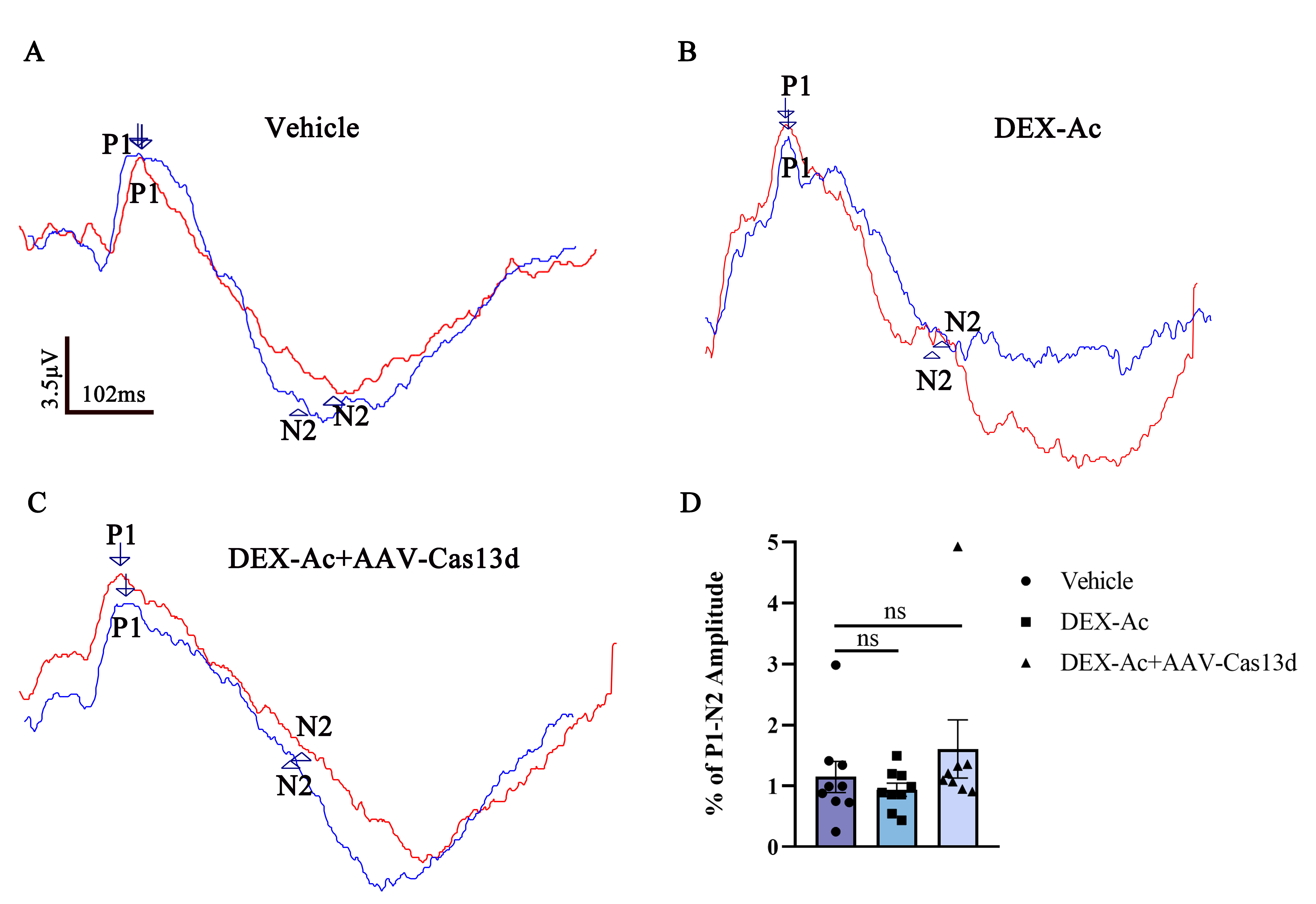


**Supplementary Figure 5. Functional analysis of AAV-Cas13d treatment in an ocular hypertension mouse model.** (A-C) Representative wave forms of PERG at baseline. Blue traces represent 1x PBS treated eyes; Red traces represent Vehicle-, DEX-Ac- or DEX-Ac+ AAV-Cas13d-treated eyes. (D) Quantification of P1-N2 amplitude of PERG four weeks after intravitreal injection. *n>*=8 per group.

**Supplementary Table 1: Sequences of all target sites used in this study.**

| Sites | Sequence (5’ to 3’) |
| --- | --- |
| hCA2-gRNA1 | AGCACAATCCAGGTCACACATTCCAGAAGA |
| hCA2-gRNA2 | GCGCCAGTTGTCCACCATCAGTTCTTCGGG |
| hAQP1-gRNA1 | ATGATGTACATGAGGGCACGGAAGATGCTG |
| hAQP1-gRNA2 | GCCAGTGTAGTCAATAGCCAGGAGGTGTCC |
| mCA2-gRNA1 | TTTATCATAAGATATGAGCAGAGGCTGTAG |
| mCA2-gRNA2 | ATCGAAGTTAGCAAAGGCCGCACGCTTCCC |
| mCA2-gRNA3 | CACAGCTTTTCCAAAGTCCCCATATTTGGT |
| mAQP1-gRNA1 | AAGTCATAGATGAGCACTGCCAGGGCACCC |
| mAQP1-gRNA2 | AGTGCCAATGATCTCAATGCCCAGGCCCTG |
| mAQP1-gRNA3 | ATGCTCAAACCAAAGGCCAGCGACACCTTC |
| mAQP1-gRNA4 | AGAACGCACAGTACCAGCTGCAGAGTGCCA |
| mAQP1-gRNA5 | CCAGTGTAGTCAATCGCCAGCAGGTGTCCA |

| Name | Sequence (5’ to 3’) |
| --- | --- |
| hAQP1 -qPCR-F1 | GCTGGTACTGTGCGTTCT |
| hAQP1 -qPCR-R1 | TGTAGTCAATCGCCAGCAG |
| hCA2-qPCR-F1 | CTGGAAACTTGGACTACTGGAC |
| hCA2-qPCR-R1 | GTACGGAAATGAGACATCTGCT |
| hGapdh-F | GTGTTCCTACCCCCAATGTG |
| hGapdh-R | TAGCCCAAGATGCCCTTCAG |
| mAQP1 -qPCR-F1 | GCTGGTACTGTGCGTTCT |
| mAQP1 -qPCR-R1 | TGTAGTCAATCGCCAGCAG |
| mAQP1 -qPCR-F2 | GGCGATTGACTACACTGGCT |
| mAQP1 -qPCR-R2 | ACTGGTCCACACCTTCATGC |
| mCA2-qPCR-F1 | AGCGAGCAGATGTCTCATTTC |
| mCA2-qPCR-R1 | GTTGTCCACCATCGCTTCTT |
| mCA2-qPCR-F2 | TGCGGCCTTTGCTAACTTC |
| mCA2-qPCR-R2 | GCTGACAGTAATGGGCTCCC |
| mGapdh-F | CCGTAGACAAAATGGTGAAGGT |
| mGapdh-R | CGTGAGTGGAGTCATACTGGAA |

**Supplementary Table 2: Primers used for qPCR test in cell lines or mouse tissue.**
